# Single-Cell and Spatial Insights into Oral Squamous Cell Carcinoma: Towards Cell-State-Targeted Immunotherapy

**DOI:** 10.32604/or.2026.091624

**Published:** 2026-10-28

**Authors:** Yang Chen, Lirong Hou

**Affiliations:** 1 Department of Stomatology, Stomatological Hospital of Southern Medical University, Guangzhou, China; 2 Department of Stomatology, Xianyang Central Hospital, Xianyang, China

**Keywords:** Oral squamous cell carcinoma, single-cell transcriptomics, spatial transcriptomics, tumor microenvironment, cancer-associated fibroblasts, cancer immunotherapy

## Abstract

Oral squamous cell carcinoma (OSCC) remains associated with substantial morbidity and mortality, and only a subset of patients derive durable benefit from immune-checkpoint blockade. Single-cell and spatial approaches reveal cellular states and tissue organization that bulk profiling cannot resolve. These findings suggest that therapeutic responses may depend on both cellular function and the local interactions that sustain it. This narrative review integrates single-cell transcriptomic, spatial, functional and clinical studies of the OSCC microenvironment. We distinguish OSCC-specific findings from broader head and neck squamous cell carcinoma and pan-cancer evidence. Computational inference, tissue association, experimental perturbation and clinical validation are considered separately. Malignant, myeloid, fibroblast, vascular and lymphoid states form spatially organized niches whose composition varies across anatomical sites and treatment settings. We examine their relevance to immune escape and chemotherapy resistance and assess strategies directed at specific cell states and intercellular circuits. Although selected circuits have functional support in experimental models, many spatial signatures remain observational and lack clinical validation. Clinical translation will require reproducible tissue assays, causal testing of candidate interactions and independent biomarker validation. Practical considerations include marker-panel selection, spatial scoring, tissue availability and the feasibility of sampling primary tumors and metastatic sites before, during and after treatment. Prospective studies must also determine whether biomarker-guided treatment improves outcomes, rather than assuming that a spatial association predicts therapeutic benefit. This review aims to critically synthesize current evidence, identify key knowledge gaps and define requirements for translating cell-state and spatial-niche insights into selective immunotherapeutic strategies for OSCC.

## 1 Introduction

Oral squamous cell carcinoma (OSCC) develops within a structurally and immunologically complex barrier tissue [1,2]. Surgery remains central to localized disease, whereas recurrent, metastatic and unresectable tumors carry substantial morbidity and mortality [2]. Nivolumab and pembrolizumab have improved treatment options for recurrent or metastatic head and neck squamous cell carcinoma (HNSCC), including oral-cavity cancers [3,4]. These randomized HNSCC trials establish clinical benefit in their enrolled populations; they do not validate selection by the cell-state or spatial-niche signatures discussed here. Many patients fail to respond or subsequently develop progression [5]. The cellular ecosystem may contribute to these outcomes, but its causal role must be tested rather than inferred from a resistance-associated signature.

Conventional histopathology, immunohistochemistry and bulk transcriptomic profiling established the prognostic relevance of tumor-infiltrating lymphocytes, macrophages, fibroblasts and immune-checkpoint molecules in OSCC [6]. However, these approaches largely measure cellular abundance or spatially averaged molecular signals. They consequently obscure several biologically important distinctions [7]. Macrophages occupying the same tumor can execute inflammatory, phagocytic, tissue-remodeling or immunoregulatory programs; fibroblasts can form physical barriers to lymphocyte infiltration but can also participate in stromal structures associated with productive antitumor immunity; and a tumor containing numerous CD8^+^ T cells can remain functionally immunosuppressed if those cells are terminally dysfunctional, spatially excluded from malignant cells or embedded within suppressive myeloid niches [8,9]. Moreover, cells sampled from the tumor core cannot necessarily be assumed to have the same functional state as phenotypically related cells at the invasive front, in draining lymph nodes or within metastatic deposits.

Single-cell technologies have begun to resolve these ambiguities [10]. An early landmark analysis of HNSCC demonstrated substantial inter- and intratumoral heterogeneity among malignant epithelial cells and identified a partial epithelial-to-mesenchymal transition (p-EMT) program enriched at the leading edge of primary tumors, where malignant cells interact extensively with cancer-associated fibroblasts (CAFs) [11]. Subsequent OSCC-specific studies have extended this framework from cellular heterogeneity to spatial organization. Integrated single-cell and spatial transcriptomic profiling of human papillomavirus (HPV)-negative OSCC demonstrated that tumor-core and leading-edge regions contain distinct malignant transcriptional programs, neighboring cell populations and ligand–receptor networks [12]. Analysis across matched normal mucosa, dysplastic lesions and early OSCC further showed that stromal and immune remodeling begins before overt invasion, including the emergence of specialized fibroblast, monocyte and regulatory T-cell states [13]. Thus, cellular heterogeneity is not merely a property of established tumors; it evolves alongside malignant transformation.

Spatial profiling reconnects cellular state with tissue architecture. Dissociative single-cell sequencing identifies coexisting states but usually cannot establish whether they interact *in situ*. In OSCC, integrated spatial analyses have identified distinct tumor-core and leading-edge programs [12], metabolically organized fibroblast regions [14], treatment-associated stromal niches [15], proliferating lymphatic endothelium at invasive margins [16], and stage-associated differences in the distribution of myeloid-derived suppressor cells (MDSCs) [17]. Each observation has a different evidentiary scope: tissue localization and clinical association do not, by themselves, establish that a particular interaction causes immune dysfunction or treatment resistance.

Accordingly, the analytical unit relevant to therapy is shifting from cell abundance to cell state, from cell state to cell–cell interaction, and ultimately from interaction to spatial niche. This distinction has important therapeutic consequences. Eliminating an entire cellular lineage may remove both tumor-promoting and tumor-restraining states, whereas selectively disrupting a disease-associated state or the molecular circuit that sustains a suppressive niche might preserve beneficial functions while improving antitumor immunity. The therapeutic unit in OSCC should therefore increasingly be viewed not as an isolated cell type, but as a disease-associated cellular state embedded within a spatially organized multicellular niche. In this Review, we use single-cell and spatial studies to define these niches and consider how their emerging biology could inform more selective, cell-targeted immunotherapy.

Throughout this review, OSCC-specific evidence refers to cohorts explicitly restricted to oral-cavity squamous carcinomas. Broader HNSCC cohorts, HPV-associated oropharyngeal studies and pan-cancer findings are identified separately. We also distinguish computational communication inference, spatial or clinical association, functional perturbation in experimental systems, and clinical treatment evidence. These categories are complementary and do not constitute a simple progression from a marker to an approved therapy. This review aims to synthesize evidence on OSCC cellular states and spatial niches, assess their therapeutic relevance, and identify priorities for mechanistic validation and clinical translation.

## 2 The OSCC Microenvironment through Single-Cell and Spatial Lenses

Single-cell profiling has expanded the cellular taxonomy of OSCC, but increasingly granular clusters alone do not explain tumor behavior. OSCC-specific analyses connect malignant-cell plasticity, stromal remodeling, immune dysfunction and vascular organization within defined compartments [12,14,15,18]. Complementary broader HNSCC studies provide mechanistic hypotheses that require confirmation in oral-cavity cohorts [19]. Anatomical and treatment context should therefore accompany each state description.

### 2.1 Malignant Cell Plasticity Establishes Spatial Tumor Architecture

Malignant epithelial heterogeneity provides an organizational scaffold for the tumor ecosystem. Single-cell analysis of oral-cavity tumors revealed coexisting differentiation, stress, hypoxic, proliferative and invasive programs within individual lesions [11]. These are within-patient state differences, rather than evidence that every tumor has one stable molecular identity. The recurrence of a program across cohorts does not establish that its prevalence or therapeutic significance is uniform across OSCC populations.

The partial epithelial-to-mesenchymal transition (p-EMT) program described by Puram and colleagues illustrates this distinction [11]. Malignant cells retained epithelial features while expressing selected invasion-associated programs, including laminin subunit gamma 2 (LAMC2), laminin subunit beta 3 (LAMB3) and podoplanin (PDPN), with p-EMT localized preferentially to the leading edge. The single-cell discovery cohort comprised oral-cavity HNSCC, whereas subsequent associations with pathological features were extended using broader The Cancer Genome Atlas HNSCC data. Thus, the anatomical scope of the discovery and validation analyses should be distinguished. Independent OSCC spatial work supports the association between malignant state and tumor position [12], but neither a p-EMT score nor tumor budding alone establishes a therapeutic dependency.

Arora and colleagues integrated spatial transcriptomics with single-cell data from HPV-negative OSCC and resolved different tumor-core and leading-edge programs [12]. The compartments differed in malignant transcription, neighboring populations and predicted ligand–receptor interactions. Leading-edge programs showed similarities across cancer types, whereas tumor-core programs were more tissue specific. These observations identify the invasive interface as a candidate site of coordinated malignant and stromal adaptation; they do not establish that every neighboring component is required for dissemination.

Traditional pathological observations are compatible with this model. Tumor budding, conventionally defined as isolated malignant cells or small cell clusters at the invasive front, is associated with lymph-node metastasis and unfavorable outcomes in OSCC [20]. Earlier histological studies interpreted budding largely through an epithelial-to-mesenchymal transition (EMT) framework, whereas emerging spatial data suggest that it may represent a dynamic invasive phenotype maintained through local interactions with the surrounding microenvironment. The relationship should nevertheless be interpreted cautiously: p-EMT signatures, tumor budding and metastatic competence overlap biologically but are not interchangeable measurements.

OSCC spatial profiling has associated regional metabolic programs with stromal and immune organization [14]. Such associations motivate testing how nutrient availability, redox conditions and paracrine signals influence neighboring cells. They do not show that every metabolically adapted region is chemotherapy resistant. Whether hypoxia, metabolic stress and reciprocal niche signaling stabilize a resistant malignant state should be tested with treatment-specific perturbation and spatial measurements.

These studies argue against viewing malignant heterogeneity as cellular noise. Instead, malignant cell states create spatially distinct ecological demands. Invasive cells require matrix remodeling and migration-permissive stroma; metabolically adapted cells reshape nutrient and signaling gradients; and metastatic cells must interact with lymphatic and immune compartments. Malignant heterogeneity therefore provides the spatial scaffold on which distinct immune and stromal niches are assembled.

### 2.2 Myeloid State Transitions Couple Tumor Progression to Immune Suppression

Myeloid cells illustrate particularly clearly why single-cell resolution requires a conceptual shift away from broad lineage labels. Macrophages in solid tumors have traditionally been divided into classically activated (M1)-like and alternatively activated (M2)-like populations [21]. Although this framework remains useful for describing experimentally polarized extremes, human OSCC macrophages occupy a substantially broader and more continuous spectrum of transcriptional states. Their functional identity is shaped by tumor stage, anatomical location, stromal signals, metabolic conditions and treatment exposure [22].

Single-cell studies have identified macrophage programs characterized by complement-associated genes such as complement C1q C chain (C1QC), extracellular-matrix and lipid-associated programs including secreted phosphoprotein 1 (SPP1), and additional inflammatory or stress-adapted states [23]. These labels should not be interpreted as immutable macrophage subtypes: C1QC and SPP1 identify recurrent transcriptional programs across cancers, but their biological effects depend on tissue and context. The emerging therapeutic question is therefore not whether macrophages are globally beneficial or detrimental, but which macrophage states dominate a particular OSCC niche and which upstream signals maintain them [24].

OSCC-specific evidence supports context-dependent myeloid states. Profiling of precancerous oral lesions identified an immune-inhibitory inhibin subunit beta A (INHBA)-expressing monocyte population, together with apolipoprotein E (APOE)/neuregulin 1 (NRG1)-associated macrophages and altered vascular endothelial growth factor (VEGF) signaling during progression toward early OSCC [13]. The INHBA population in that study should not be relabeled as macrophages. In established OSCC, fibroblast activation protein (FAP)-positive fibroblasts were reported to promote C1QC-positive macrophage accumulation through Wnt family member 2 (WNT2) and β-catenin-associated signaling [25]. These observations motivate testing stromal control of macrophage function; marker expression alone does not establish suppressive activity or selective susceptibility to treatment.

Metastatic sites provide further evidence of anatomical differences. In OSCC patients receiving neoadjuvant treatment, protein kinase R-like endoplasmic reticulum kinase (PERK)-positive macrophages were associated with resistant lymph-node metastases [18]. The same study used functional models to investigate macrophage-mediated matrix remodeling and impaired T-cell cytotoxicity. Human association and model-based mechanism are complementary, but they do not establish PERK-positive macrophages as a prospectively validated treatment-selection biomarker. A primary-tumor biopsy may fail to represent the nodal ecosystem.

In a 2026 OSCC study integrating single-cell and spatial transcriptomics, Li and colleagues observed stage-associated differences in MDSC localization [17]. Earlier-stage samples showed greater tumor-core localization, whereas advanced-stage samples showed marginal enrichment near functionally impaired CD8-positive T cells. These human comparisons were cross-sectional and did not track individual cells moving between compartments. Computational ligand–receptor analysis implicated tumor-derived annexin A1 (ANXA1) and formyl peptide receptor signaling. Pharmacological formyl peptide receptor 2 (FPR2) inhibition with WRW4, including combination with anti-programmed cell death protein 1 (PD-1) treatment, provided functional support in syngeneic models. This intervention supports a preclinical hypothesis but does not isolate every proposed ligand–receptor step or establish efficacy in patients [17].

Neutrophil-like and granulocytic suppressor states probably contribute to the same continuum, although their precise taxonomy in human OSCC remains less resolved than that of macrophages [26]. Dissociation, short cellular lifespan and transcriptional sensitivity can also complicate neutrophil characterization by conventional single-cell RNA sequencing. These technical limitations should temper strong claims regarding discrete neutrophil subtypes. Nevertheless, the emerging picture is one of myeloid plasticity across both state and space, in which inflammatory recruitment can evolve into persistent immunoregulation as tumors progress [27].

Collectively, these observations make the M1/M2 dichotomy insufficient as the principal framework for OSCC. Macrophage and MDSC biology is better understood along axes of recruitment, differentiation, metabolic adaptation, effector function and anatomical localization. This view also changes therapeutic reasoning: indiscriminate macrophage depletion risks eliminating antigen-presenting or phagocytic populations together with suppressive cells, whereas targeting the signals that create pathogenic states, such as fibroblast-to-macrophage or tumor-to-MDSC circuits, may provide greater specificity.

### 2.3 Fibrovascular Niches Govern Immune Access and Exclusion

Stromal cells help organize the physical and signaling environment surrounding malignant cells. Pan-cancer single-cell analyses identify heterogeneous fibroblast programs [28], and OSCC treatment-associated profiling demonstrates stromal states with different clinical associations [15]. The labels myofibroblastic, inflammatory and matrix-remodeling describe recurring programs rather than fixed, universally protumor lineages. Their meaning and therapeutic relevance must be assessed in the anatomical and treatment context in which they were measured.

Spatial analysis of OSCC has shown that metabolic geography can influence fibroblast identity. Liu and colleagues integrated single-cell and spatial transcriptomic data and identified metabolically distinct regions associated with transformation toward inflammatory CAF states [14]. Their analysis linked tumor metabolism with stromal remodeling and an immunosuppressive microenvironment, illustrating that CAF identity can be conditioned by local metabolic constraints rather than being fixed across the tumor. Together with the leading-edge architecture described by Arora and colleagues [12], these observations support a model in which fibroblasts near invasive tumor states acquire programs adapted to matrix remodeling, inflammatory signaling and reciprocal communication with malignant and myeloid cells.

Recent mechanistic data further connect fibroblast state to immune dysfunction. The FAP^+^ fibroblast–WNT2–C1QC^+^ macrophage circuit described in OSCC provides one example: fibroblast-derived signaling altered macrophage state, which was in turn associated with exhausted T-cell phenotypes [25]. Such findings shift the interpretation of CAFs from isolated stromal effectors to organizers of multicellular immune-suppressive circuits. Dense extracellular matrix, altered matrix composition and mechanical signaling may additionally limit lymphocyte motility and access to malignant cells, although the relative contribution of physical exclusion versus soluble immunoregulation is likely to vary between tumors [19,29].

Paired profiling of 22 patients with OSCC from a randomized phase II neoadjuvant trial identified stromal states associated with different treatment responses [15]. Responding tumors after immunochemotherapy were enriched for selectin P (SELP)-positive high endothelial venules (HEVs) and apolipoprotein D (APOD)-positive myofibroblastic CAFs, whereas stathmin 1 (STMN1)-positive capillary endothelial cells and myogenic factor 5 (MYF5)-positive muscle satellite cells were linked to less favorable neighborhoods. These are clinical correlative findings from a translational subset, rather than randomized tests of stromal targeting. They support caution about indiscriminate CAF depletion but do not prove that inducing APOD-positive CAFs or HEVs improves clinical outcome.

FAP is useful for identifying activated stroma and for imaging or targeting, but it does not uniquely identify one pathogenic fibroblast state [30]. Transforming growth factor beta (TGFβ) signaling also participates in tissue repair and homeostasis. Stromal normalization or selective state modulation is therefore a therapeutic hypothesis requiring context-specific testing, rather than an established OSCC treatment recommendation [31,32].

Vascular states add a complementary dimension. Specialized endothelial structures can support immune trafficking, whereas abnormal vascular networks may contribute to hypoxia or impaired leukocyte entry. In the neoadjuvant OSCC study, SELP-positive HEVs and STMN1-positive capillary endothelial populations had different associations with immune organization and response [15]. This is clinical correlative support for a distinction between vascular states, rather than a direct test of their respective functions.

A 2026 spatial single-cell study of oral-cavity cancers examined more than 700,000 cells and associated lymphatic endothelial cells expressing marker of proliferation Ki-67 (KI67) and prospero homeobox 1 (PROX1) at the invasive margin with recurrence in early-stage OSCC [16]. The association was evaluated in an independent cohort. This supports prognostic relevance in the studied setting, rather than a claim that targeting these cells prevents metastasis or predicts benefit from a specific intervention.

The emerging fibrovascular landscape is therefore bidirectional. Some stromal and vascular states support invasion, immune exclusion and metastatic dissemination; others facilitate immune-cell recruitment and potentially treatment response. The relevant question is not whether stroma or vasculature should be eliminated, but which fibrovascular states should be suppressed, preserved or therapeutically induced.

### 2.4 Lymphoid State and Position Define Immune Competence

The same principle applies to lymphocytes: their presence does not establish their functional capacity. Conventional measurements of tumor-infiltrating lymphocytes implicitly assume that greater immune infiltration reflects stronger antitumor immunity. Single-cell analyses of OSCC demonstrate instead that infiltrating T cells occupy a spectrum ranging from naïve or memory-like states to activated cytotoxic, regulatory and dysfunctional programs [33]. The composition of this spectrum varies between patients and is further shaped by spatial context.

For CD8-positive T cells, differentiation state, antigen specificity, clonal expansion and localization provide complementary information. General tumor-immunology studies distinguish progenitor-like populations capable of expansion from more terminally dysfunctional states [34,35]. These findings are not all OSCC-specific. Transcriptional phenotype and clonal expansion alone do not establish tumor-antigen recognition; paired T-cell receptor sequencing and antigen-specific functional assays are needed to interpret therapeutic competence. HPV-specific T-cell analyses in HNSCC illustrate this distinction [36].

OSCC-specific studies support the importance of these distinctions. Single-cell analysis of tumor-infiltrating T-cell populations identified marked heterogeneity among cluster of differentiation 4 (CD4)^+^ and CD8^+^ compartments and heterogeneous activation and dysfunction states across tumors [33]. In precancerous lesions, Sun and colleagues identified regulatory T-cell remodeling, including a tumor necrosis factor receptor superfamily member 4 (TNFRSF4)-associated regulatory population that emerged within the evolving immunosuppressive microenvironment [13]. These observations suggest that regulatory adaptation is already underway before fully invasive disease is established.

Activated tumor-associated regulatory T (Treg) cells can suppress cytotoxic immunity through inhibitory receptors, cytokines and competition for growth factors [37]. Systemic Treg depletion risks disrupting peripheral immune tolerance. Useful targets would therefore distinguish tumor-adapted regulatory states from circulating or homeostatic Tregs. TNFRSF4/CD134 costimulatory receptor (OX40)-associated states have been described in oral lesions, whereas evidence for C-C motif chemokine receptor 8 (CCR8) targeting derives from several solid tumors and broader HNSCC studies [13,38,39]. Their specificity and therapeutic window should be established separately in OSCC.

Natural killer (NK) cells and other cytotoxic innate-like lymphocytes are less extensively mapped in OSCC than conventional T cells. Available studies support heterogeneity in cytotoxic activity and spatial distribution, but current OSCC datasets do not yet justify a stable taxonomy of discrete NK states comparable with that emerging in larger pan-cancer atlases. Their activity is nevertheless likely to be influenced by many of the same suppressive features that constrain T cells, including myeloid-derived cytokines, TGFβ-rich stromal environments, hypoxia and physical exclusion. This is particularly relevant for future NK-cell-based therapies, in which the presence of NK cells in a tumor should not be equated with preserved killing capacity [40].

Spatial association reinforces these distinctions. Advanced OSCC samples show CD8-positive T cells near MDSC-rich margins [17], whereas the neoadjuvant study associated SELP-positive HEVs and APOD-positive myofibroblastic CAFs with favorable immune organization [15]. These comparisons identify candidate suppressive and immune-supportive neighborhoods. They do not prove that tumors with similar CD8-positive cell counts have equivalent tumor reactivity, nor that a neighborhood remains stable during subsequent treatment or recurrence.

Thus, immune-cell abundance does not equal immune competence. A more informative description integrates cell state, clonality, localization and neighborhood. This framework is likely to be particularly important for biomarkers intended to guide checkpoint blockade or cellular immunotherapy, because therapeutic success requires not only the presence of cytotoxic cells but also their ability to reach, recognize and remain functional adjacent to malignant targets.

### 2.5 Multicellular Niches Are the Functional Units of OSCC

When considered together, single-cell and spatial studies suggest that the major biological behaviors of OSCC are generated by recurring multicellular configurations rather than individual cell types acting independently. These configurations are not yet standardized pathological entities, and their boundaries will vary across technologies and cohorts. Nevertheless, several provisional niche archetypes provide a useful framework for integrating current evidence (Fig. 1).

**Figure 1:**
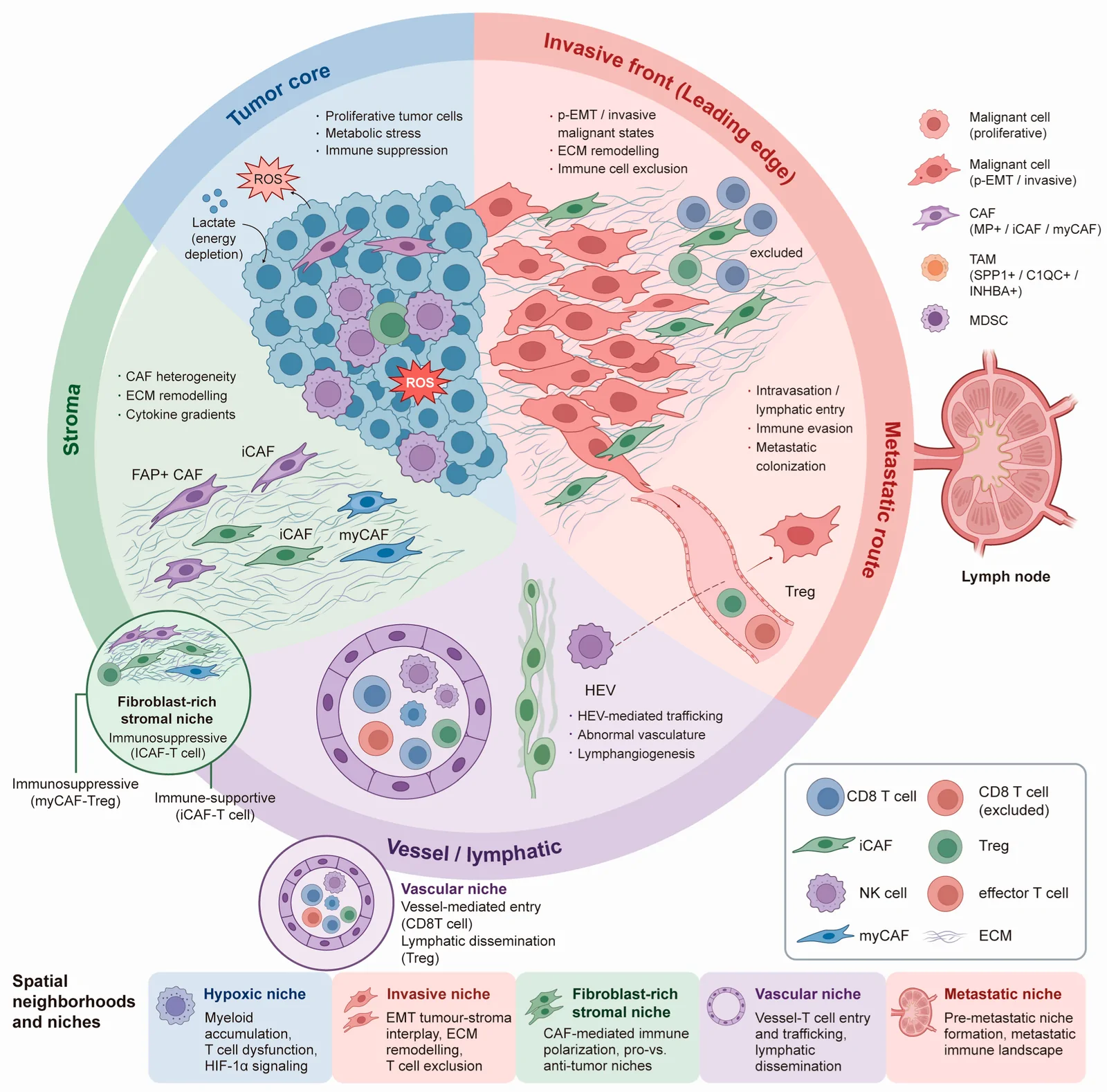
Cellular states and spatial niches in oral squamous cell carcinoma. The schematic organizes representative malignant, stromal, myeloid and lymphoid programs across the tumor core, invasive front, stroma, vascular and lymphatic interfaces, and regional lymph nodes. The lower panels illustrate provisional niche archetypes. Cell labels represent context-dependent programs, not fixed functional lineages or validated treatment-selection categories. In particular, the INHBA-positive monocyte population reported in oral precancer should be distinguished from INHBA-positive macrophages described in other contexts. Fibroblast-mediated immune support or suppression also depends on the specific state and setting. Abbreviations: OSCC, oral squamous cell carcinoma; p-EMT, partial epithelial-to-mesenchymal transition; MP, matrix-producing; CAF, cancer-associated fibroblast; FAP, fibroblast activation protein; iCAF, inflammatory cancer-associated fibroblast; myCAF, myofibroblastic cancer-associated fibroblast; TAM, tumor-associated macrophage; MDSC, myeloid-derived suppressor cell; ECM, extracellular matrix; HEV, high endothelial venule; treg, regulatory T cell; NK, natural killer cell; ROS, reactive oxygen species; SPP1, secreted phosphoprotein 1; C1QC, complement C1q C chain; INHBA, inhibin subunit beta a; HIF-1α, hypoxia-inducible factor 1 alpha; CD8, cluster of differentiation 8; EMT, epithelial-to-mesenchymal transition.

An invasive niche can be conceptualized as leading-edge malignant cells with p-EMT or related programs beside matrix-remodeling fibroblasts and recruited myeloid cells. Histological budding has adverse clinicopathological associations in OSCC [20], while single-cell and spatial studies identify malignant programs and neighboring populations at the invasive interface [11,12]. Their coexistence suggests interactions to test; it does not prove that every budding focus has the same fibroblast or myeloid dependency.

A myeloid-suppressive niche is characterized by suppressive myeloid states near functionally impaired lymphocytes. Stage-associated MDSC enrichment at advanced OSCC margins is one example [17]. Fibroblast-associated C1QC-positive macrophages and PERK-positive macrophages in resistant nodal metastases illustrate distinct candidate circuits at different sites [18,25]. A fibrotic immune-excluded niche contains matrix-rich stroma that may separate cytotoxic cells from malignant targets. Conversely, SELP-positive HEVs and APOD-positive myofibroblastic CAFs were associated with favorable responses to neoadjuvant immunochemotherapy [15]. A metastatic-access niche includes invasive malignant cells and lymphatic endothelium; margin-associated KI67-positive, PROX1-positive lymphatic endothelial cells have prognostic associations [16]. These archetypes summarize complementary observations and should not be treated as interchangeable, standardized clinical categories.

Niche categories remain working biological models. Single-cell measurements are sensitive to dissociation, sampling and annotation, while spatial platforms differ in cellular resolution and tissue coverage. Ligand–receptor inference identifies plausible communication and spatial proximity identifies potential contact; neither establishes a causal signaling dependency. Stronger conclusions require concordant molecular localization, cell-specific perturbation and independent validation. Table 1 summarizes the anatomical source, evidence type and current validation level of representative states and circuits.

### 2.6 Cellular Heterogeneity and Chemotherapy Resistance

Conventional chemotherapy adds a selective pressure that differs from immune-checkpoint blockade. Single-cell profiling of cisplatin-sensitive and progressively resistant OSCC cell lines identified brain acid soluble protein 1 (BASP1)-enriched resistant and transitional states. BASP1 knockdown reversed mesenchymal features and restored cisplatin-induced cell death [41]. This supports a functional dependency in experimental OSCC cells, not a validated patient biomarker or a demonstrated resistant spatial niche.

Stromal signaling can also modify drug sensitivity. CAF preparations from OSCC secreted midkine, and conditioned medium protected oral carcinoma cells from cisplatin. Midkine neutralization and perturbation of ANRIL (antisense noncoding RNA in the INK4 locus), a long noncoding RNA, supported a paracrine resistance mechanism [42]. Its spatial organization and therapeutic utility in patients remain unvalidated. Profiling of residual tumor and tumor bed from one patient after induction chemotherapy identified heterogeneous epithelial, fibroblast and macrophage programs and predicted interactions [43]. Without a paired pretreatment sample or additional patients, that observation cannot establish treatment-induced changes or a reproducible resistance signature.

Immune exclusion and chemotherapy resistance may coexist, but require separate tests. Hypoxia or matrix could affect local drug exposure; this spatial hypothesis requires validation. Regional drug measurements and organoid–stromal perturbation, combined with pretreatment, on-treatment and residual or recurrent sampling, could distinguish selection from adaptation. Immunochemotherapy response cannot isolate the chemotherapy component [15,44].

**Table 1:** Representative cellular states and niches with evidence scope and validation level.

State or Niche	Markers or Pathway	Biological Role	Disease Source	Supporting Evidence	Therapeutic Implication	Current Validation
Invasive malignant program	LAMC2, LAMB3, PDPN; p-EMT	Invasion-associated epithelial plasticity	Oral-cavity discovery; broader HNSCC TCGA analysis	Single-cell profiling and leading-edge localization [11]; independent OSCC spatial architecture [12]	Candidate malignant–stromal interface intervention	Descriptive and prognostic association; no validated treatment-selection assay
FAP-positive fibroblast–macrophage circuit	FAP–WNT2–C1QC; β-catenin	Stromal regulation of macrophage accumulation and T-cell dysfunction	OSCC tissue and experimental systems	Profiling, tissue association and functional pathway experiments [25]	Test selective fibroblast-to-macrophage signaling inhibition	Preclinical mechanistic support; no demonstrated patient benefit
MDSC-rich tumor margin	ANXA1–FPR signaling; FPR2	Spatial myeloid suppression near cytotoxic cells	OSCC human samples and syngeneic models	Cross-sectional spatial/ligand–receptor inference; WRW4 inhibition with anti-PD-1 [17]	Test FPR2-directed combinations	Pharmacological support in models; ligand/cell specificity and clinical efficacy unvalidated
Invasive-front fibroblast niche	PLAU–ITGB1–TGFβ; COL11A1	Fibroblast activation and local immune suppression	OSCC tissues and experimental models	Spatial inference plus gene knockdown, co-culture and *in vivo* intervention [45]	Test selective pathway or fibroblast-state modulation	Direct preclinical perturbation; clinical selectivity and efficacy unestablished
Resistant nodal myeloid niche	PERK-positive macrophages	Matrix remodeling and impaired nodal T-cell function	OSCC lymph-node metastases and models	Paired anatomical clinical association; functional macrophage/PERK experiments [18]	Test PERK-pathway modulation in nodal disease	Preclinical support; no prospective predictive assay
Treatment-associated immune-supportive stroma	SELP-positive HEVs; APOD-positive myCAFs	Immune recruitment associated with treatment response	OSCC; 22-patient translational trial subset	Paired single-cell profiles; post-treatment spatial clinical correlates [15]	Preserve candidate immune-supportive structures; test baseline signatures	Correlative human evidence; causality and predictive utility unvalidated
Proliferating invasive-margin lymphatics	KI67; PROX1	Lymphatic proliferation associated with recurrence	Oral-cavity/OSCC clinical cohorts	Spatial protein measurements and independent prognostic validation [16]	Candidate spatial risk biomarker	Prognostic association; no intervention-specific prediction or causal target validation
Tumor-adapted regulatory lymphoid state	CCR8; TNFRSF4/OX40	Local suppression of cytotoxic immunity	Oral-lesion TNFRSF4 data; broader HNSCC/solid-tumor CCR8 data	OSCC profiling [13]; anti-CCR8 preclinical/*ex vivo* experiments [39]	Investigational selective tumor-Treg depletion	Mechanistic support outside OSCC-specific efficacy trials; clinical benefit unestablished
Progenitor-like cytotoxic state	TCF1; PD-1; antigen-specific TCR	Retention of proliferative and differentiation capacity	General tumor models and HPV-associated HNSCC	Functional antigen-specific and checkpoint-response experiments [34,36]	Preserve or expand responsive T-cell states	Biological principle; transferability to HPV-negative OSCC requires testing
Suppressive myeloid/cellular therapy interface	CXCR1/2; granulocytic MDSCs	Suppression of therapeutic T- or NK-cell activity	Oral/head and neck experimental models	SX-682 trafficking blockade with T- or NK-cell treatment [46,47]	Investigational niche conditioning for cellular therapy	Direct preclinical activity; clinical efficacy unestablished
Macrophage phagocytosis circuit	CD47–SIRPα; CD11b/CD18–ICAM1	Control of tumor-cell adhesion and engulfment	HNSCC experimental systems	Receptor blockade, adhesion and phagocytosis experiments [48]	Investigational cetuximab plus phagocytosis checkpoint blockade	Functional model evidence; OSCC-specific efficacy unestablished
Cisplatin-resistant malignant state	BASP1; mesenchymal plasticity	Cisplatin survival and cell plasticity	OSCC cell-line models	Single-cell analysis and BASP1 knockdown restoring cisplatin sensitivity [41]	Investigational malignant-state sensitization	Functional cell-model evidence; spatial niche and patient prediction unvalidated
Stromal chemotherapy-protection circuit	Midkine–ANRIL	Paracrine protection from cisplatin-induced death	OSCC-derived CAFs and oral carcinoma cell models	Conditioned-medium, midkine neutralization and ANRIL perturbation experiments [42]	Investigational paracrine resistance blockade	Direct preclinical support; spatial and clinical validation absent

Abbreviations: OSCC, oral squamous cell carcinoma; HNSCC, head and neck squamous cell carcinoma; TCGA, The Cancer Genome Atlas; p-EMT, partial epithelial-to-mesenchymal transition; LAMC2, laminin subunit gamma 2; LAMB3, laminin subunit beta 3; PDPN, podoplanin; FAP, fibroblast activation protein; WNT2, Wnt family member 2; C1QC, complement C1q C chain; MDSC, myeloid-derived suppressor cell; ANXA1, annexin A1; FPR, formyl peptide receptor; FPR2, formyl peptide receptor 2; PD-1, programmed cell death protein 1; PLAU, plasminogen activator urokinase; ITGB1, integrin subunit beta 1; TGFβ, transforming growth factor beta; COL11A1, collagen type XI alpha 1 chain; PERK, protein kinase R-like endoplasmic reticulum kinase; SELP, selectin P; HEV, high endothelial venule; APOD, apolipoprotein D; myCAF, myofibroblastic cancer-associated fibroblast; KI67, marker of proliferation Ki-67; PROX1, prospero homeobox 1; CCR8, C-C motif chemokine receptor 8; TNFRSF4, tumor necrosis factor receptor superfamily member 4; OX40, CD134 costimulatory receptor; Treg, regulatory T cell; TCF1, T-cell factor 1; TCR, T-cell receptor; HPV, human papillomavirus; CXCR1/2, C-X-C motif chemokine receptors 1 and 2; NK, natural killer cell; CD47, cluster of differentiation 47; SIRPα, signal regulatory protein alpha; CD11b/CD18, cluster of differentiation 11b/18; ICAM1, intercellular adhesion molecule 1; BASP1, brain acid soluble protein 1; CAF, cancer-associated fibroblast; ANRIL, antisense noncoding RNA in the INK4 locus. All therapeutic implications in the table remain investigational unless clinical treatment evidence is explicitly stated. Markers do not by themselves establish lineage, function or target selectivity.

## 3 Single-Cell and Spatial Guidance for Cell-Targeted Immunotherapy

Single-cell and spatial profiling can identify states associated with resistance, their locations and candidate interactions. Therapeutic interpretation requires an evidence hierarchy. Computational or spatial associations generate hypotheses; perturbation experiments test necessity or sufficiency in a defined model; paired human biopsies can demonstrate pharmacodynamic changes; and clinical benefit requires appropriately controlled trials. In this section, cell-state- and circuit-directed treatments are discussed as research strategies unless clinical efficacy is explicitly established. Model-based functional support should not be equated with a validated OSCC therapy.

### 3.1 Reprogramming the Myeloid Compartment Rather than Eliminating Macrophages

The marked heterogeneity of the myeloid compartment makes it an instructive example of how single-cell analysis can change therapeutic reasoning. Rather than conforming to a stable M1–M2 dichotomy, macrophages and monocyte-derived populations occupy overlapping inflammatory, phagocytic, lipid-associated, matrix-remodeling and immunoregulatory states [49,50]. Consequently, indiscriminate macrophage depletion could remove both tumor-supporting populations and cells capable of antigen presentation, phagocytosis or immune activation. The therapeutic goal should instead be to prevent the establishment of suppressive myeloid states, redirect established macrophages toward antitumor functions, or restore effector mechanisms that tumors actively inhibit [51].

One strategy is to limit recruitment of suppressive myeloid cells. Several chemokine and growth-factor pathways can contribute to this process. Tumor-derived C-C motif chemokine ligand 2 (CCL2) has long been implicated in monocyte recruitment in HNSCC; experimental work demonstrated a reciprocal CCL2–epidermal growth factor (EGF) circuit in which tumor cells recruited macrophages that subsequently promoted malignant-cell motility and invasion [52]. Colony-stimulating factor 1 (CSF1)–colony-stimulating factor 1 receptor (CSF1R) signaling represents another major determinant of macrophage survival and differentiation, although experience across solid tumors indicates that CSF1R blockade does not uniformly convert immunologically resistant tumors into responsive ones. Redundant recruitment pathways and compensatory myeloid programs probably limit the efficacy of targeting any single axis.

The C-X-C motif chemokine ligand (CXCL)–C-X-C motif chemokine receptor (CXCR) system provides a more direct connection between myeloid trafficking and cellular immunotherapy. In syngeneic oral and lung squamous carcinoma models, C-X-C motif chemokine receptor 2 (CXCR2)^+^ polymorphonuclear MDSCs were potent suppressors of tumor-infiltrating lymphocytes. Pharmacological inhibition of C-X-C motif chemokine receptors 1 and 2 (CXCR1/2) with SX-682 reduced intratumoral MDSC trafficking and enhanced both checkpoint blockade and adoptively transferred T-cell activity [46]. A subsequent head and neck cancer study similarly showed that inhibiting MDSC trafficking enhanced NK-cell immunotherapy, supporting the principle that cytotoxic lymphocytes may fail not because they lack intrinsic activity but because suppressive myeloid populations occupy the same therapeutic niche [47]. These studies remain predominantly preclinical, but they provide an important mechanistic rationale for integrating myeloid-state and trafficking biomarkers into trials of T- or NK-cell-directed therapies.

The ANXA1–formyl peptide receptor (FPR) axis provides an OSCC-specific example. Human profiling identified stage-associated MDSC distributions and predicted communication with tumor cells; it did not establish longitudinal migration [17]. FPR2 inhibition reduced cluster of differentiation 11b (CD11b)-positive myeloid-cell accumulation and enhanced anti-PD-1 activity in syngeneic experiments. Thus, the proposed target has computational, spatial and pharmacological support, but requires ligand-specific and cell-specific perturbation, rescue experiments and independent validation before the full circuit is regarded as causal or therapeutically established.

A second strategy is to reprogram tumor-supportive macrophage states. Recurrent macrophage programs characterized by SPP1 or C1QC have been observed across multiple solid tumors, including HNSCC and OSCC, but these markers should not be treated as binary therapeutic labels. SPP1^+^ macrophages in HNSCC have been associated with aggressive disease and were reported to promote malignant-cell proliferation and migration through tumor necrosis factor (TNF) and interleukin 1 beta (IL-1β)-dependent signaling [53]. More recent spatially resolved multi-omics analyses of the HNSCC leading edge identified C1QC^+^ and SPP1^+^ macrophages as components of a self-reinforcing invasive ecosystem containing fibroblasts and cytotoxic lymphocytes [54]. These observations suggest that macrophage state is linked to the signaling and metabolic properties of the surrounding niche rather than solely to ontogeny.

Innate stimulation provides another reprogramming strategy. In a phase Ib neoadjuvant HNSCC study, motolimod plus cetuximab altered suppressive myeloid populations, but this pharmacodynamic activity did not establish general therapeutic benefit [55]. In a carcinogen-induced mouse model of oral premalignancy, cluster of differentiation 40 (CD40) agonism reduced progression and enhanced CD8-positive T-cell responses [56]. This is preclinical prevention evidence from oral lesions, rather than a treatment result in established human OSCC. Such findings support testing myeloid activation while emphasizing the gap between model-based activity and durable clinical benefit.

Macrophage-mediated phagocytosis is a complementary strategy. In HNSCC experimental systems, cluster of differentiation 47 (CD47)–signal regulatory protein alpha (SIRPα) blockade enhanced cetuximab-associated phagocytosis. It also promoted CD11b/cluster of differentiation 18 (CD18)-dependent macrophage adhesion to intercellular adhesion molecule 1 (ICAM1)-expressing cancer cells [48]. These functional observations provide a mechanism in the tested HNSCC models; OSCC-specific efficacy and a clinically useful selection biomarker remain unestablished.

This example also illustrates why phagocytosis-directed treatment is likely to work best as a context-dependent combination rather than as an isolated “eat-me” signal. Fragment crystallizable (Fc) receptor engagement by tumor-directed antibodies can provide a pro-phagocytic input, whereas inhibitory receptors such as SIRPα impose a countervailing brake. A recent framework emphasizes that therapeutic phagocytosis is governed by the balance among multiple activating and inhibitory receptors and cautions that first-generation phagocytosis-checkpoint therapies have shown variable efficacy and toxicity in clinical development [57]. Thus, single-cell and spatial profiling could ultimately be used to identify tumors in which macrophages are physically positioned to engage malignant cells, retain phagocytic machinery and are restrained by a defined checkpoint. The goal is not to create more macrophages, but to restore productive macrophage function in the correct cellular neighborhood.

### 3.2 Rewiring Fibroblast and Matrix Programs to Restore Immune Access

OSCC profiling identifies fibroblast states with different associations to treatment response [15]. Broader head and neck cancer literature provides complementary biological frameworks [31,32]. Candidate stromal strategies include suppressing tumor-promoting programs, interrupting fibroblast–immune communication and preserving immune-supportive structures. These objectives require functional testing and should not be inferred from the CAF label alone.

Recent OSCC work linked invasive-front epithelial cells to collagen type XI alpha 1 chain (COL11A1)-positive fibroblasts through a plasminogen activator urokinase (PLAU)–integrin subunit beta 1 (ITGB1)–TGFβ pathway [45]. Spatial profiling and computational communication analysis were complemented by PLAU, ITGB1 and COL11A1 knockdown, co-culture and *in vivo* intervention. These perturbations provide direct functional support for the proposed pathway and fibroblast phenotype in the tested systems. They do not establish COL11A1 as a clinically selective drug target, nor prove that every interaction in the epithelial–fibroblast–Treg niche is necessary in human tumors. Independent models and prospective clinical testing remain required. In immunocompetent mice, the anti-PD-1 combination targeted integrin beta1 rather than selectively depleting COL11A1-positive fibroblasts [45].

The FAP-positive fibroblast–WNT2–C1QC-positive macrophage circuit illustrates why a communication node may matter more than a lineage label [25]. Co-culture and *in vivo* experiments supported stromal regulation of macrophage accumulation, and FAP inhibition reduced the C1QC-positive compartment and improved anti-PD-1 activity in the tested systems. A WNT2/β-catenin-associated mechanism was reported, but these findings should not be described as cell-state-exclusive depletion or as proof that every FAP-positive fibroblast is harmful. Selective interruption of the circuit remains a preclinical therapeutic strategy requiring independent validation and assessment of beneficial stromal and myeloid functions.

Paired single-cell profiling and post-treatment spatial measurements identified APOD-positive myofibroblastic CAFs and SELP-positive HEVs in OSCC neighborhoods associated with favorable immune infiltration and response [15]. These findings do not establish fibroblast causality or show that inducing the state improves outcome. They motivate experiments that weaken matrix and signaling programs associated with exclusion while assessing whether useful immune-entry structures are preserved.

This view also brings vascular biology into CAF-directed therapy. Dense matrix, elevated tissue pressure and abnormal vasculature are interconnected determinants of lymphocyte access. Consequently, successful remodeling may require simultaneous correction of matrix architecture and endothelial function rather than destruction of either compartment [58]. HEV-associated immune recruitment is particularly relevant to cellular immunotherapy because transferred T or NK cells must reach the tumor before receptor specificity or cytotoxic potency can matter. Single-cell-guided stromal interventions should therefore be evaluated not only by reductions in CAF abundance but also by whether they alter lymphocyte trafficking, tumor–immune contact and the spatial distribution of functional effector cells.

### 3.3 Selectively Dismantling Regulatory Lymphoid States

Treg cells present perhaps the clearest example of the tension between antitumor efficacy and tissue homeostasis [59]. Their suppressive activity can protect tumors from cytotoxic immunity, yet the same lineage is indispensable for preventing pathological autoimmunity. Broad Treg depletion is therefore unlikely to provide an acceptable therapeutic solution [60]. The key question is instead: can tumor-adapted Treg states be selectively removed or functionally disrupted while preserving systemic immune tolerance?

Tumor-associated Tregs can express cytotoxic T-lymphocyte-associated protein 4 (CTLA4), CCR8, OX40/TNFRSF4 and T-cell immunoreceptor with immunoglobulin and immunoreceptor tyrosine-based inhibitory motif domains (TIGIT), but these are not automatically tumor-exclusive markers. TNFRSF4-associated Treg states have been observed during progression from oral precancer to OSCC [13]. Evidence for the anti-CCR8 antibody CHS-114 includes preclinical and *ex vivo* HNSCC depletion of CCR8-positive tumor Tregs [39]. This supports preferential targeting in those experimental settings. It does not establish clinical efficacy, a validated OSCC companion biomarker or complete preservation of systemic tolerance.

CTLA4, OX40 and TIGIT present related but distinct therapeutic opportunities [61]. Their expression is shared with activated conventional T cells, so the consequences of antibody treatment depend on receptor biology, Fc-mediated depletion, agonism versus antagonism and treatment context [62]. Indeed, anti-CTLA4 activity cannot be reduced simply to Treg removal, and OX40 agonism can produce different outcomes depending on whether effector or regulatory populations dominate the responding compartment. Single-cell profiling may therefore be most valuable not as a companion test for a single surface marker, but as a way to define the relative abundance, activation state and spatial position of suppressive versus effector CD4^+^ populations before treatment [63,64].

Metabolic adaptation offers another potential source of selectivity. Experimental evidence outside OSCC shows that lactate can sustain tumor-infiltrating Tregs [65]. Its transferability to OSCC, and the therapeutic window for interfering with lactate or lipid use, require direct testing [38]. Metabolic pathways are shared by multiple immune and stromal populations, so an intervention must demonstrate preferential disruption of suppressive states while preserving useful effector and regulatory functions.

### 3.4 Restoring Cytotoxic T- and NK-Cell Competence

Dysfunctional CD8-positive T cells include progenitor-like, transitional and more terminally differentiated populations with different expansion potential following checkpoint inhibition [34,36,66,67]. These principles derive from mechanistic studies across tumor contexts and broader HNSCC, rather than a universally validated OSCC state hierarchy. Removing suppressive neighbors will be insufficient when the remaining effector cells cannot recognize malignant targets or sustain function.

In HPV-associated HNSCC, antigen-specific analyses identified PD-1-positive, T-cell factor 1 (TCF1)-positive stem-like CD8-positive T cells alongside transitional and more terminally differentiated populations [36]. Related receptor clonotypes linked these states, and functional tests demonstrated retained proliferative and differentiation capacity. The viral antigenic context differs from conventional HPV-negative OSCC. This is a mechanistic example of why checkpoint expression alone does not establish whether a cell remains responsive to intervention, rather than a validated OSCC treatment-selection hierarchy.

Spatial context further constrains effector function. Stem-like or tumor-reactive cells may be separated from malignant targets or embedded in suppressive myeloid niches. In OSCC, high programmed death ligand 1 (PD-L1) expression on conventional dendritic cells in tumor-draining lymph nodes has been associated with poor prognosis [68]. That study supports nodal immune regulation, rather than a direct claim that OSCC nodes contain a therapeutically validated TCF1-positive reservoir. Primary and nodal immune compartments should therefore be assessed together when feasible.

Checkpoint inhibition can expand selected progenitor-like T-cell populations, but does not reliably restore all terminally dysfunctional cells [34,36]. Natural killer (NK) cells provide a complementary approach because their recognition is less dependent on conventional peptide–major histocompatibility complex (MHC) presentation [40,69]. In preclinical head and neck cancer models, CXCR1/2 inhibition with SX-682 reduced MDSC trafficking and improved NK-cell immunotherapy [47]. This supports niche modulation in the tested models, not established clinical efficacy in OSCC. Current OSCC studies do not yet define a universally reproducible NK-state taxonomy or a validated NK-directed treatment-selection test.

Collectively, these findings suggest that single-cell profiling should help distinguish which cytotoxic cells require reinvigoration, which require protection from suppressive niches and which have progressed too far toward terminal dysfunction to represent realistic therapeutic substrates. For both endogenous immunity and adoptive cell therapy, effective treatment will depend on matching cellular state with the intervention required to preserve or restore competence [70].

### 3.5 Targeting Cellular Circuits Rather than Individual Populations

Representative circuits have different levels of support. The fibroblast–WNT2–macrophage pathway [25], ANXA1–FPR signaling [17], and PLAU–ITGB1–TGFβ pathway [45] combine profiling with functional experiments, although the perturbations and unresolved steps differ. SPP1-associated macrophage mechanisms include broader HNSCC evidence [53,54]. By comparison, SELP-positive HEV/APOD-positive CAF neighborhoods are principally clinical associations from an OSCC translational cohort [15]. Fig. 2 presents a prospective research workflow for testing these hypotheses, rather than an established treatment algorithm.

**Figure 2:**
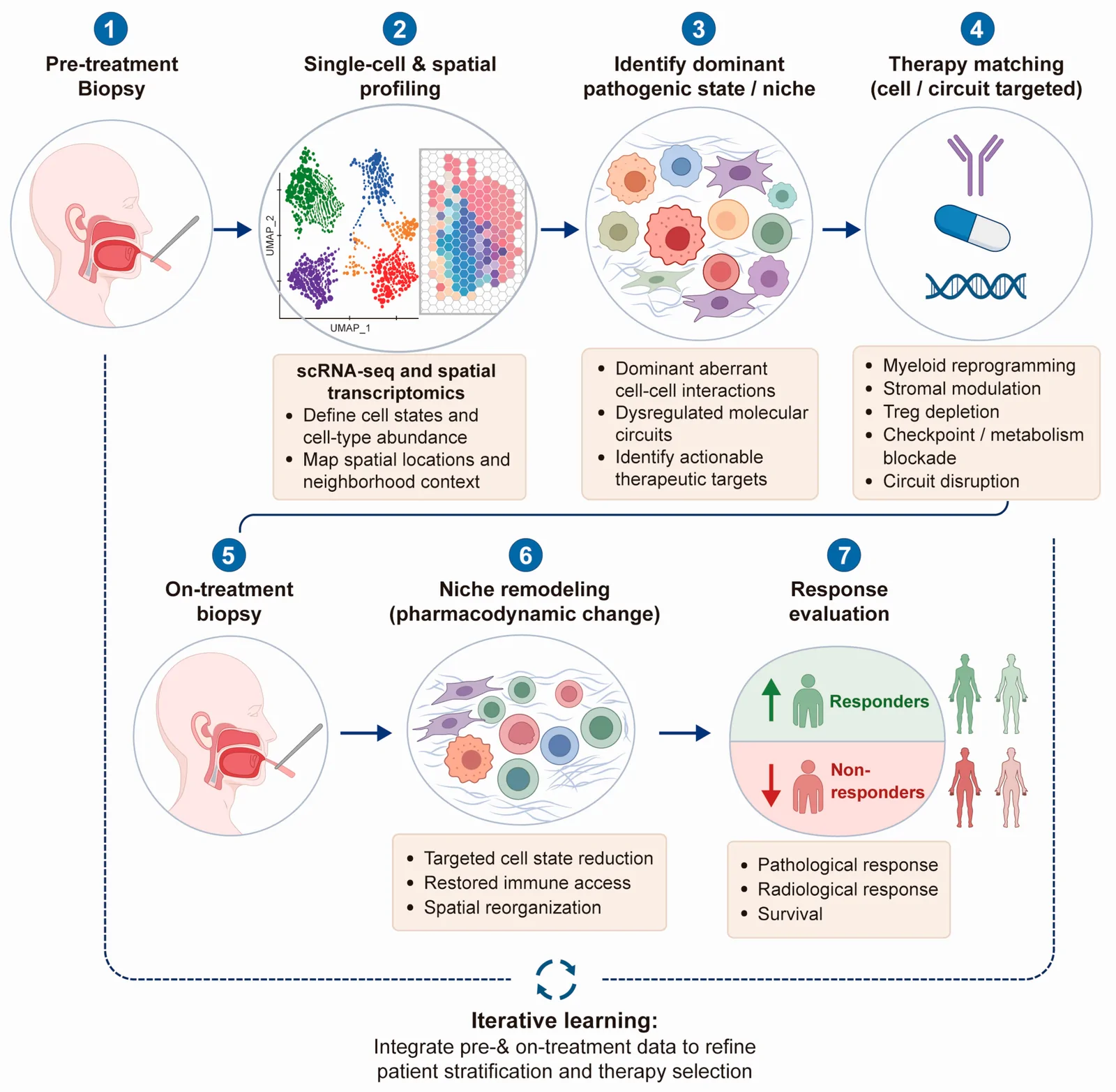
Proposed research workflow for cell-state- and circuit-directed immunotherapy in oral squamous cell carcinoma. Pretreatment sampling and single-cell or spatial profiling identify candidate states, neighborhoods and communication pathways. Functional validation and biomarker qualification are required before these features can guide therapy in prospective trials. On-treatment sampling evaluates target engagement and niche remodeling, alongside pathological response, radiological response and survival. The learning cycle uses paired measurements to refine hypotheses and trial stratification. Therapy matching and the depicted interventions remain investigational; the schematic does not imply a validated clinical decision rule. Abbreviations: scRNA-seq, single-cell RNA sequencing; UMAP, uniform manifold approximation and projection; treg, regulatory T cell.

The relevant intervention may therefore be a shared upstream node rather than simultaneous depletion of several lineages. Spatially resolved multi-omics in HNSCC identified a leading-edge ecosystem containing tumor, fibroblast and macrophage programs, with experimental interventions supporting the potential to disrupt its signaling and metabolic reinforcement [54]. This is broader HNSCC functional evidence and requires separate evaluation in OSCC. The number of interacting populations in a spatial map should not, by itself, determine the number of drugs in a combination.

An ecosystem-matched trial would identify a candidate niche, test its functional dependencies and evaluate a targeted intervention. Proposed nodes include suppressive myeloid recruitment, fibroblast signaling, tumor-adapted Tregs and impaired vascular access. Treatment evaluation should ask whether the intended state changes and whether productive tumor–immune contacts are restored, alongside conventional outcomes [15,18]. This is a proposed development strategy, not evidence that current signatures can already assign optimal therapy.

The opportunity is to turn state and niche descriptions into experimentally testable interventions. A candidate circuit-directed strategy should demonstrate that disruption of a necessary interaction changes tumor control while preserving useful cellular functions. Its precision and clinical value must be measured rather than assumed.

A causal test must address the cell type producing a signal, the responding cell and the proposed downstream function. Cell-specific ligand or receptor knockdown, selective depletion, pathway blockade and rescue can distinguish necessity from sufficiency. Co-cultures, organotypic slices or organoids containing stromal and immune compartments can test whether an interaction changes drug sensitivity or lymphocyte killing while preserving spatial context. Spatial RNA or protein measurements establish localization; they should be paired with pathway-activity and functional assays. *In vivo* validation should assess tumor control, off-target effects and preservation of beneficial populations. Independent human cohorts and paired biopsies are then needed to test transferability and target engagement. These are recommended future tests, not experiments performed for this review.

## 4 Clinical Translations: From Cellular Biomarkers to Therapeutic Trials

The translational question is whether a measured state or niche can select treatment, demonstrate target engagement and explain resistance. Resectable OSCC offers access to pretreatment biopsies and surgical specimens after neoadjuvant therapy. This permits paired measurement, but neither accessibility nor correlation with response establishes clinical utility. The evidence below distinguishes randomized treatment comparisons, exploratory tissue correlates, early pharmacodynamic observations and prospective trial concepts.

### 4.1 Single-Cell and Spatial Biomarkers in OSCC Immunotherapy Trials

The randomized phase II NCT04649476 trial compared neoadjuvant camrelizumab alone with camrelizumab plus docetaxel, cisplatin and fluorouracil in 68 patients with resectable, locally advanced OSCC [44]. The combination produced deeper pathological responses, supporting its activity in the studied setting. The trial did not randomize patients according to a spatial-niche classifier and therefore does not validate niche-guided treatment selection.

The translational study analyzed 40 tumor specimens from 22 trial participants, with spatial transcriptomics performed on post-treatment specimens from 11 patients [15]. Favorable responses after immunochemotherapy were associated with enrichment of SELP-positive HEVs and APOD-positive myofibroblastic CAFs and depletion of STMN1-positive capillary endothelial cells. Spatial measurements linked these states to immune recruitment and antigen-presentation-related features. These are exploratory clinical correlates from a small subset, with no intermediate on-treatment sampling or in-depth functional validation. They cannot isolate chemotherapy-specific mechanisms or prove that the stromal changes caused response.

Paired sampling can also expose anatomical discordance that would otherwise be obscured by analysis of the primary tumor alone. In patients with OSCC treated with neoadjuvant immunotherapy or immunochemotherapy, Zhang and colleagues observed less favorable pathological responses in lymph-node metastases than in corresponding primary tumors and identified PERK^+^ macrophages as a feature of resistant nodal ecosystems [18]. Functional experiments implicated these macrophages in extracellular-matrix remodeling and impaired T-cell cytotoxicity, and PERK inhibition enhanced anti-PD-1 activity in experimental models. Although PERK^+^ macrophages require validation as a prospective clinical biomarker, this study highlights a broader trial-design issue: a biomarker obtained from the primary tumor may fail to represent the biology of metastatic disease.

These studies motivate longitudinal ecosystem biomarkers. A candidate should be measured before therapy, tracked during treatment and evaluated against pathological or clinical outcomes. An association in a post-treatment surgical specimen may reflect treatment exposure or response rather than a predictive baseline feature. Establishing prediction requires a prospectively specified test and evidence that outcomes differ according to both biomarker status and treatment, rather than a responder-versus-nonresponder comparison alone.

### 4.2 Clinical Targeting of Suppressive Lymphoid and Myeloid Compartments

The first clinical attempts to translate cell-state biology are now emerging, although most derive from broader HNSCC rather than OSCC-specific cohorts. Targeting CCR8^+^ intratumoral Treg cells is one example. CHS-114 (tagmokitug) is an afucosylated anti-CCR8 antibody designed to preferentially deplete CCR8-expressing tumor Tregs through Fc-dependent effector mechanisms [39]. Preclinical and *ex vivo* studies demonstrated selective depletion of CCR8^+^ Tregs while largely sparing CCR8-negative Tregs and conventional effector T cells, providing a mechanistic rationale for tumor-selective rather than systemic Treg depletion [39].

CHS-114 is being evaluated in the phase I NCT05635643 program, including broader HNSCC cohorts [71]. The preclinical and *ex vivo* depletion data provide a rationale for this program [39], rather than evidence of clinical efficacy. OSCC-specific activity, durable benefit and a validated selection threshold remain unestablished. Clinical target engagement and antitumor outcomes must be assessed separately.

Clinical experience with myeloid-directed therapy provides an instructive counterexample. In a neoadjuvant phase Ib study of 14 patients with untreated HNSCC, the Toll-like receptor 8 (TLR8) agonist motolimod combined with cetuximab reduced intratumoral MDSCs, increased inflammatory monocyte features, enhanced CD8^+^ T-cell infiltration, and produced multiple favorable immune biomarker changes [55]. These pharmacodynamic findings suggested that suppressive myeloid states were modifiable in patients. However, the subsequent randomized Active8 trial, which added motolimod to platinum–fluorouracil–cetuximab in recurrent or metastatic HNSCC, did not improve progression-free or overall survival in the intention-to-treat population [72]. An apparent signal in HPV-positive disease was hypothesis-generating rather than definitive.

This discordance between compelling immune modulation and negative population-level clinical efficacy is a useful translational lesson. Demonstrating that a drug changes the myeloid compartment does not establish that the affected myeloid state is the dominant resistance mechanism in every patient. Broad myeloid stimulation administered to an unselected population may therefore dilute benefit in the subset whose tumors are genuinely myeloid dependent. Future trials should ask not only whether a myeloid-directed intervention is biologically active, but which pretreatment myeloid ecosystem predicts dependence on that intervention.

### 4.3 Stromal and TGFβ-Directed Strategies

The clinical development of TGFβ-directed therapy illustrates a similar tension between strong biological rationale and heterogeneous therapeutic results. TGFβ can contribute to fibroblast activation, extracellular-matrix remodeling, immune exclusion, and suppression of lymphocyte function, making simultaneous inhibition of TGFβ and checkpoint pathways attractive. Bintrafusp alfa, a bifunctional fusion protein combining a TGFβ trap with PD-L1 blockade, was evaluated in a phase I cohort of 32 heavily pretreated patients with recurrent or metastatic HNSCC [73]. The confirmed objective response rate was 13%, demonstrating clinical activity but falling short of establishing that generalized TGFβ trapping could overcome resistance in an unselected HNSCC population.

Ficerafusp alfa (BCA101) combines epidermal growth factor receptor (EGFR) targeting with TGFβ sequestration. Two-year results from a phase I/Ib HNSCC expansion cohort reported antitumor activity with pembrolizumab, but the study was nonrandomized and cannot establish superiority over standard treatment [74]. A randomized phase II/III study, FORTIFI-HN01 (NCT06788990), is designed to test the addition of ficerafusp alfa to pembrolizumab in a broader recurrent or metastatic HNSCC population [75]. Oral-cavity eligibility does not establish an OSCC-specific effect, and the contribution of any spatial biomarker requires separate validation.

FAP-directed imaging illustrates the distinction between measuring a target and validating treatment directed at it. Studies in OSCC demonstrated uptake of FAP inhibitors in oral tumors and cervical metastases [76] and evaluated gallium-68 fibroblast activation protein inhibitor (FAPI) positron emission tomography/computed tomography for nodal staging [77]. These imaging observations do not establish that FAP marks a uniform immunosuppressive CAF state or that FAP-directed therapy improves immunotherapy outcomes.

However, the ability to visualize FAP-rich stroma does not mean that FAP identifies a universal pathogenic CAF population. Indeed, an HNSCC study reported discordance between FAP transcript abundance and protein expression and found limited evidence that FAP^+^ CAFs themselves drove checkpoint resistance in the tested models [78]. This observation is consistent with the single-cell evidence discussed above: fibroblast states associated with favorable and unfavorable immunity can coexist within OSCC. FAP imaging may therefore become valuable for mapping stromal burden or selecting FAP-directed theranostic approaches, but single-cell-defined CAF states are biologically more informative than single-marker CAF targeting when the therapeutic objective is immune remodeling.

### 4.4 Building Biomarker-Enriched Cell-Targeted Trials

Future OSCC trials could prospectively stratify or enrich patients using reproducible candidate states, such as suppressive myeloid, regulatory lymphoid or immune-excluded stromal programs. These categories should be defined before enrollment and evaluated with assay procedures and thresholds fixed in advance. An exploratory cluster annotation is insufficient for assigning treatment [79,80].

Critically, treatment should be followed by an on-treatment or surgical specimen whenever clinically feasible. The primary pharmacodynamic question would then be whether the intended biological perturbation occurred: were CCR8^+^ Tregs selectively depleted; did suppressive macrophages change state; did an immune-excluding fibroblast program regress; did effector cells move across a previously restrictive stromal boundary; or did an HEV-associated immune-supportive neighborhood emerge? These measurements are conceptually distinct from tumor shrinkage and can identify mechanistic success even when radiographic response is incomplete, or, conversely, mechanistic failure despite administration of an apparently appropriate drug [80,81].

Spatial position should be evaluated alongside abundance. Fewer circulating MDSCs may not mean removal of a suppressive tumor-margin neighborhood, and more CD8-positive cells may not mean productive tumor contact. Mechanistic endpoints could integrate state abundance, spatial proximity and relevant clonotype dynamics with pathological response and conventional outcomes [15,18]. These measurements would help distinguish an invalid hypothesis from inadequate target engagement or an inappropriate study population; their clinical utility remains to be established.

### 4.5 Practical Biomarker Implementation and Longitudinal Validation

Clinical implementation could follow a discovery-to-assay pathway rather than require whole-transcriptome single-cell sequencing for every patient. Candidate states discovered by high-resolution profiling can be reduced to small marker panels compatible with formalin-fixed, paraffin-embedded (FFPE) tissue. Multiplex immunohistochemistry or immunofluorescence, RNA *in situ* hybridization, targeted transcript panels and digital pathology are practical approaches to evaluate these features. A spatial assay should retain the feature that made the niche informative, such as the anatomical compartment or proximity of suppressive myeloid cells to cytotoxic lymphocytes, rather than measuring marker positivity alone. FAP-directed imaging offers a complementary means of mapping stromal burden in OSCC, but it does not identify a particular pathogenic CAF state [76,77]. The choice of assay should balance tissue availability, turnaround time, cost and access to pathology expertise.

Translation requires analytical validity before clinical interpretation. Fixation, specimen handling, marker specificity, image segmentation, compartment definitions and between-laboratory reproducibility should be standardized. A reduced signature, scoring algorithm and decision threshold should be fixed after discovery and tested in independent OSCC cohorts. Clinical validation should evaluate performance across disease stage, treatment setting and biopsy location and determine whether the assay adds useful information to established clinical and pathological factors [80]. Prognostic association, pharmacodynamic target engagement and prediction of treatment benefit are distinct endpoints. A predictive claim requires evidence that benefit differs according to biomarker status, ideally through a prespecified biomarker-by-treatment interaction in a randomized trial; association with response in a single treatment arm is insufficient.

Longitudinal and multisite sampling is essential because neither cell state nor anatomical context can be assumed to remain stable. When clinically feasible, prospective studies should sample the primary tumor core and invasive margin separately, pair these with involved regional lymph nodes, and obtain pretreatment, early on-treatment and surgical specimens. Recurrence or metastatic-progression biopsies should reassess the current niche before a baseline result is carried forward. Discordant primary-tumor and nodal responses in OSCC [18], together with treatment-associated stromal remodeling [15], support this need. Core and margin samples provide spatial information, while repeated time points distinguish pre-existing states from treatment-associated changes. Such designs can test whether a baseline niche persists, shifts location or is replaced by a new resistance program.

These studies should use consistent processing and annotation across time points and record anatomical site, prior therapy, dose and sampling interval. Analysis must respect the patient as the biological unit: thousands of cells or tissue neighborhoods from one tumor do not constitute independent patient replicates. Within-patient comparisons can separate changes in state abundance from altered marker expression or spatial relocation, although destructive biopsies cannot demonstrate that the same individual cells were followed over time. Missing biopsies and limited tissue coverage should be reported, and conclusions should be restricted to the sampled sites and clinical setting. Until temporal and spatial transferability is demonstrated, a baseline niche should be regarded as a context-dependent candidate biomarker rather than a durable guide to all subsequent treatment.

## 5 Conclusions and Outlook: From Cell Atlases to Precision Ecosystem Therapy

Single-cell and spatial technologies have expanded the description of OSCC. The next challenge is to determine which states drive disease, which reflect adaptation and which can be safely targeted. Increasingly detailed cluster annotations should lead to testable hypotheses rather than increasingly confident therapeutic claims.

Transcriptional identity alone does not establish function. An adverse macrophage signature may indicate a causal suppressor state, a stromal response or a marker of aggressive disease. Likewise, CAF enrichment in responding tumors does not prove that the fibroblasts mediate treatment sensitivity. Candidate states require orthogonal localization, selective perturbation and, ultimately, pharmacodynamic and clinical testing.

The biological effect of a state may differ between tumor core, invasive front, lymph node and distant lesion. Its position determines access to malignant cells, local metabolic conditions and interacting partners. The therapeutic unit is therefore a state within a location and a set of interactions, rather than a marker alone. Its persistence over time must be measured instead of assumed.

This framework could inform trials of myeloid reprogramming, stromal modulation, selective Treg targeting or restoration of cytotoxic function. When several suppressive populations depend on a common pathway, circuit-directed combinations may be preferable to broad lineage depletion. These are hypotheses for prospective testing, rather than a clinically validated classification or treatment system.

Current OSCC cohorts are often small, spatial measurements cover limited tissue areas, and many dependencies have principally preclinical support. Independent cohorts, reproducible state definitions, longitudinal multi-site sampling and clinically scalable assays are needed. Progress also requires distinguishing prognosis from treatment prediction and demonstrating that an assay-guided strategy improves patient outcomes.

High-resolution discovery should ultimately produce reproducible biomarkers that can be measured in routine tissue. The aim is to identify a biologically justified intervention and verify that it changes the intended state or interaction. Until prospective clinical utility is established, cell-state and spatial-niche profiling should support research and trial development rather than determine routine OSCC treatment.

## Data Availability

Not applicable.

## Abbreviations

**Term**	**Interpretation**
ANRIL	Antisense noncoding RNA in the INK4 locus
ANXA1	Annexin A1
APOD	Apolipoprotein D
APOE	Apolipoprotein E
BASP1	Brain acid soluble protein 1
C1QC	Complement C1q C chain
CAFs	Cancer-associated fibroblasts
CCL2	C-C motif chemokine ligand 2
CCR8	C-C motif chemokine receptor 8
CD11b	Cluster of differentiation 11b
CD18	Cluster of differentiation 18
CD4	Cluster of differentiation 4
CD40	Cluster of differentiation 40
CD47	Cluster of differentiation 47
CD8	Cluster of differentiation 8
COL11A1	Collagen type XI alpha 1 chain
CSF1	Colony-stimulating factor 1
CSF1R	Colony-stimulating factor 1 receptor
CT	Computed tomography
CTLA4	Cytotoxic T-lymphocyte-associated protein 4
CXCL	C-X-C motif chemokine ligand
CXCR	C-X-C motif chemokine receptor
CXCR1/2	C-X-C motif chemokine receptors 1 and 2
CXCR2	C-X-C motif chemokine receptor 2
ECM	Extracellular matrix
EGF	Epidermal growth factor
EGFR	Epidermal growth factor receptor
EMT	Epithelial-to-mesenchymal transition
FAP	Fibroblast activation protein
FAPI	Fibroblast activation protein inhibitor
FFPE	Formalin-fixed, paraffin-embedded
FPR	Formyl peptide receptor
FPR2	Formyl peptide receptor 2
Fc	Fragment crystallizable
HEV	High endothelial venule
HLA	Human leukocyte antigen
HNSCC	Head and neck squamous cell carcinoma
HPV	Human papillomavirus
ICAM1	Intercellular adhesion molecule 1
IL-1β	Interleukin 1 beta
INHBA	Inhibin subunit beta A
ITGB1	Integrin subunit beta 1
KI67	Marker of proliferation Ki-67
LAMB3	Laminin subunit beta 3
LAMC2	Laminin subunit gamma 2
MDSCs	Myeloid-derived suppressor cells
MHC	Major histocompatibility complex
MYF5	Myogenic factor 5
NK	Natural killer cell
NRG1	Neuregulin 1
OSCC	Oral squamous cell carcinoma
OX40	CD134 costimulatory receptor
PD-1	Programmed cell death protein 1
PD-L1	Programmed death ligand 1
PDPN	Podoplanin
PERK	Protein kinase R-like endoplasmic reticulum kinase
PET	Positron emission tomography
PLAU	Plasminogen activator urokinase
PROX1	Prospero homeobox 1
RNA	Ribonucleic acid
SELP	Selectin P
SIRPα	Signal regulatory protein alpha
SPP1	Secreted phosphoprotein 1
STMN1	Stathmin 1
TCF1	T-cell factor 1
TCGA	The Cancer Genome Atlas
TCR	T-cell receptor
TGFβ	Transforming growth factor beta
TIGIT	T-cell immunoreceptor with immunoglobulin and immunoreceptor tyrosine-based inhibitory motif domains
TLR8	Toll-like receptor 8
TNF	Tumor necrosis factor
TNFRSF4	Tumor necrosis factor receptor superfamily member 4
Treg	Regulatory T cell
UMAP	Uniform manifold approximation and projection
VEGF	Vascular endothelial growth factor
WNT2	Wnt family member 2
p-EMT	Partial epithelial-to-mesenchymal transition
scRNA-seq	Single-cell RNA sequencing
